# Beyond generalist AI: purpose-built foundation models for transplantation

**DOI:** 10.3389/fmed.2026.1988867

**Published:** 2026-10-21

**Authors:** Layal Jbara, Varun Kanal, Soumita Ghosh, Praveen Manickavel, Mathula Muhundan, Samhita Mallavarapu, Venkat Bhat, Divya Sharma, Mamatha Bhat

**Affiliations:** 1 Ajmera Transplant Centre, University Health Network, Toronto, ON, Canada; 2 Schulich School of Medicine & Dentistry, Western University, London, ON, Canada; 3 Interventional Psychiatry Program, Department of Psychiatry, St. Michael’s Hospital, Unity Health Toronto, Toronto, ON, Canada; 4 Temerty Centre for Artificial Intelligence Research and Education in Medicine, University of Toronto, Toronto, ON, Canada; 5 Institute of Medical Science, Temerty Faculty of Medicine, University of Toronto, Toronto, ON, Canada; 6 Department of Psychiatry, University of Toronto, Toronto, ON, Canada; 7 Biostatistics Department, University Health Network, Toronto, ON, Canada; 8 Department of Mathematics and Statistics, York University, Toronto, ON, Canada; 9 Division of Gastroenterology and Hepatology, Department of Medicine, University of Toronto, Toronto, ON, Canada

**Keywords:** clinically grounded AI, donor–recipient–graft ecosystem, foundation models, governed deployment, longitudinal transplant continuum, multimodal integration, organ allocation, solid organ transplantation

## Abstract

Solid organ transplantation is a defining test case for clinically grounded foundation models. These models have begun to transform language, imaging, and pathology elsewhere in medicine, yet many remain built around a single patient and a static snapshot. Transplant outcomes instead arise from a dynamic donor–recipient–graft ecosystem across the care continuum, under organ scarcity and fragmented multimodal data. Published work has adapted generalist models to isolated tasks, showing component feasibility rather than transplant-native pretraining. In this Perspective, we explain why generalist medical AI may leave transplant care’s needs unmet. We define five requirements for transplant-native models: joint donor–recipient–graft representation, longitudinal continuum memory, multimodal integration, decision awareness, and continual adaptation and grounding under human oversight. We then set out a staged roadmap from data infrastructure and model development through clinical validation to governed, assistive deployment. Early use should remain bounded and assistive, judged by compatibility with transplant biology, clinical workflow, patient safety, and ethical allocation rather than scale alone.

## 1 Introduction

Solid organ transplantation has progressed considerably through advances in surgery, therapy, management and care, yet major complications including infection, rejection, and highly variable long-term survival persist (1). Unlike many other medical specialties, transplantation arises from a uniquely dual and dynamic biology: two separate biological entities whose interaction determines outcome across a pre-, peri-, and post-transplant continuum rather than a single disease event. Clinicians must synthesize a broad multimodal spectrum of data under substantial uncertainty and organ scarcity (2, 3).

Artificial Intelligence (AI) and deep learning (DL) are increasingly being applied across the solid organ transplantation care pathway (heart, kidney, liver, lung, and related organs), supporting tasks such as donor–recipient matching, survival prediction, risk stratification, early detection of graft dysfunction, and optimization of immunosuppressive therapy (4, 5). However, existing AI approaches remain narrowly task-specific and modality-constrained, primarily focusing on specific prediction tasks while failing to capture the longitudinal, multimodal, and interconnected nature of the transplant continuum. This reflects an architectural mismatch rather than a scaling gap: transplant medicine is not a static diagnostic problem but a continuously adapting ecosystem in which fragmented AI architectures are poorly suited to this structure.

Foundation models (FMs), large-scale models that learn transferable representations across diverse downstream tasks, have begun to transform language, imaging, pathology, and multimodal reasoning across medicine (6–8). Healthcare is increasingly moving toward specialized, modality-aware models rather than a single universal FM. We posit that transplantation sits beyond the conventional generalist-vs.-specialist debate: many FMs are still designed around a single patient and a static clinical snapshot, whereas transplant care requires reasoning across a donor–recipient–graft ecosystem that evolves over time. Maturing national registries, multimodal medical FMs, and the scale of clinical documentation now make transplant-native pretraining increasingly feasible. As a result, outputs that appear highly capable in general applications may remain misaligned in transplantation, where decisions must frequently be made under conditions of urgency, uncertainty, evolving patient trajectories, life-sustaining therapies, limited resources, or time-sensitive interventions in which delays can increase morbidity and mortality. Here, we use *transplant-native* to describe a foundation model whose underlying representations are developed using transplant-native data and learning objectives. Such a model would learn how linked donor, recipient, and graft characteristics evolve across the care continuum, yielding representations that can support multiple downstream tasks. It could be developed through transplant-native pretraining or substantial continued pretraining of an existing model. Fine-tuning a general medical model for one transplant task alone would not meet this definition.

The growing divide between generalist and specialized healthcare FMs highlights a central challenge for transplantation: whether generalist medical AI alone can meet the distinct clinical needs of the fields remains an open question. Transplantation should not be treated as a downstream application of general-purpose models, reduced to generalist clinical copilots, or fragmented into isolated prediction tasks. It requires transplant-native FMs designed to integrate donor–recipient interactions, longitudinal graft trajectories, immunological dynamics, multimodal diagnostics, and allocation decisions within a unified framework.

We argue that transplantation is a uniquely compelling and high-stakes setting for domain-specific FMs because performance must align not only with data patterns, but also with transplant biology, clinical workflow, ethical allocation, and rigorous validation. The field must therefore move toward clinically grounded, multimodal models that treat the donor–recipient–graft ecosystem as the unit of intelligence, learn evolving graft biology rather than static snapshots, and support the full care continuum while remaining bounded, interpretable, assistive, and clinically governed (Figure 1).

**Figure 1:**
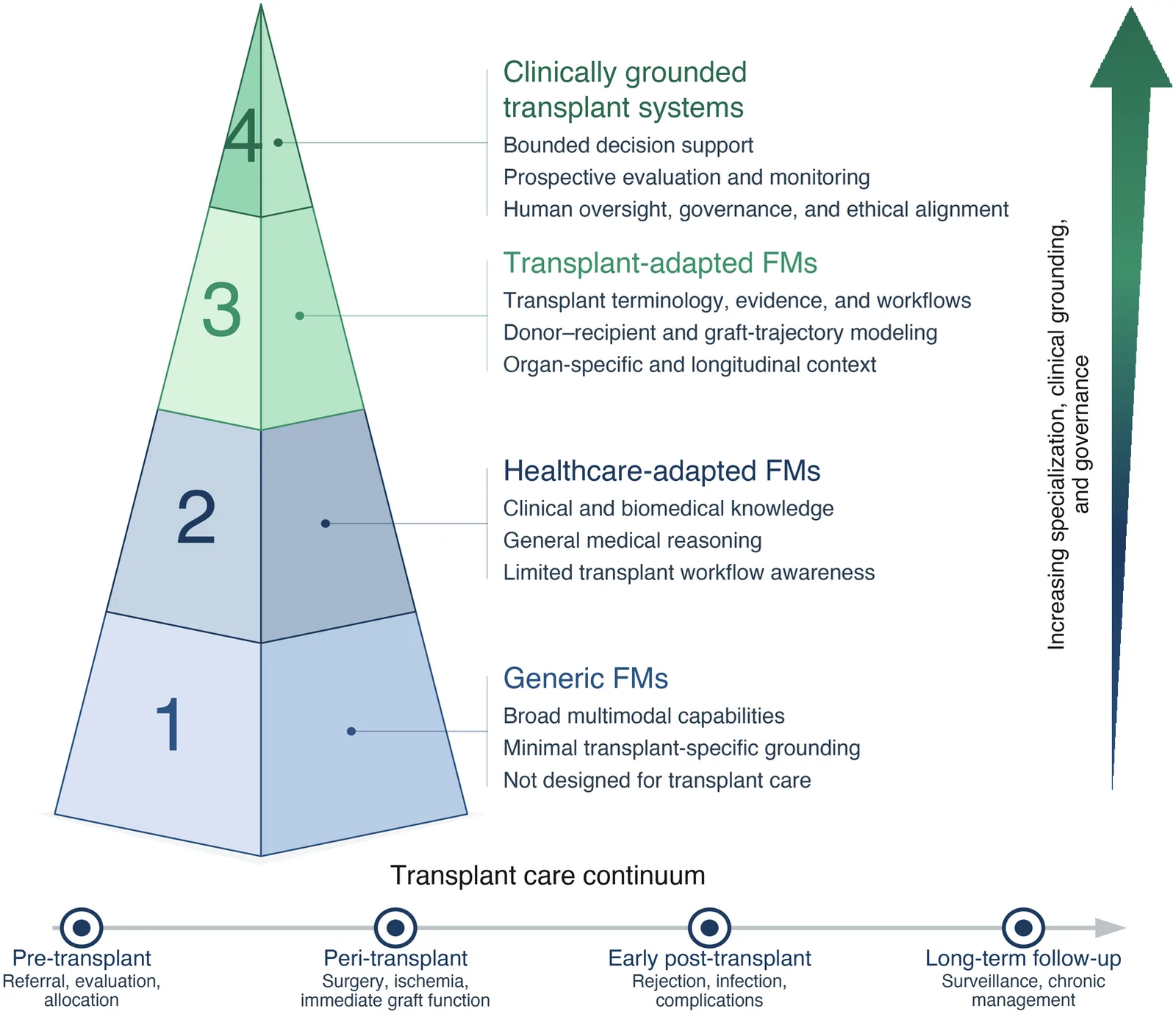
From generalist FMs to clinically grounded transplant-native systems. Progressive hierarchy from generalist FMs to healthcare-adapted models, transplant-adapted FMs, and clinically grounded transplant systems. Increasing specialization, clinical grounding, and governance are required to support decision-making across the transplant care continuum.

In this Perspective, we synthesise how FMs have been applied in transplantation, explain why generalist medical AI may be insufficiently aligned with the needs of transplant care, and propose a staged roadmap for transplant-native models.

## 2 What makes transplantation unique

While domain-specific FMs have begun to emerge across several medical specialties, their role in transplantation remains comparatively embryonic. Transplantation is structurally distinct for FM development. This uniqueness does not result from the sheer quantity and diversity of data alone, but from the very nature of the clinical challenge, wherein the outcome depends on the interplay among the donor, recipient, and graft; decisions evolve dynamically over an extended period of time; and predictions could potentially impact the distribution of scarce, life-saving organs. The above conditions give rise to certain needs that are not fully addressed by conventional patient-focused or task-oriented AI models (Supplementary Figure S1). Transplant FMs must therefore be designed around the defining characteristics of the field.

### 2.1 The transplant continuum

Transplant medicine can be generally divided into pre-transplant, peri-operative, and post-transplant phases. Each stage calls for a unique but related reasoning process depending on its specific constraints and goals. What distinguishes this continuum is that the relevance of clinical tasks changes across phases, so phase context is essential for interpreting any observation (9). Among the objectives summarised in Supplementary Table S1, organ-offer and allocation decision support under time pressure and post-transplant rejection surveillance represent the highest-value and highest-risk targets for FM development. FM development in transplantation must not only account for biological compatibility and graft-related outcomes but also fairness, psychosocial readiness, and barriers to access.

In many clinical domains, FMs can plausibly assume that the current patient chart contains the information needed for a given task. Transplant care challenges this assumption across every phase transition because signals are time-dependent and context-sensitive. For instance, the exact same lab value (such as a rising creatinine) or tissue sample result (biopsy pattern like acute tubular necrosis) means vastly different things based on the patient’s temporal trajectory. FMs optimized for static clinical snapshots, single admissions, single imaging studies, or single pathology slides may not adequately represent the temporal structure: they treat each encounter as diagnostically self-contained, whereas transplant clinicians reason across evolving physiological states in which the interpretation of each signal depends on what preceded it. Transplant FMs must instead maintain longitudinal state representations across phase transitions, continually updating interpretation as context changes. The transplant continuum, not a series of disconnected stage-specific predictions assembled post hoc, should be the unit of temporal reasoning.

### 2.2 The donor–recipient–graft ecosystem

In most medical fields, prediction concerns disease risk, treatment response, or prognosis within a single patient, a structure to which existing FMs naturally conform. Transplantation differs because its defining question is whether a specific donor organ will survive and function in a specific recipient. This outcome emerges from donor–recipient interaction, organ quality, and immunocompatibility rather than either party alone. Alloimmune risk must be managed through HLA matching, donor-specific antibody screening, crossmatching, and balanced immunosuppression (10–12). A model centered on one patient chart therefore may not fully address transplantation’s core prediction problem. Transplant FMs must treat paired biological success as the object of learning from pretraining onward.

This relationship is difficult to represent because donor and recipient data are generated and stored separately. Recipient information typically resides in local electronic health records, whereas donor and organ-quality variables are distributed across donor hospitals, organ procurement organizations, allocation systems, pathology platforms, and procurement records. Yet graft outcomes cannot be inferred from recipient characteristics or immunological compatibility alone: donor injury, organ quality, and ischemic exposure may substantially alter outcomes despite favourable matching. This fragmentation reflects transplantation’s multidisciplinary, cross-institutional structure, in which clinicians, laboratories, pathologists, social workers, and procurement organizations contribute data through incompatible systems, formats, and vocabularies (13). The biological unit determining outcome is therefore divided across records never designed to form a unified representation. Models pretrained on single-hospital data consequently lack the joint donor–recipient view required for transplantation and may not reliably reconstruct it from recipient data alone.

Allocation adds a further layer of complexity. Because donor organs are scarce, each match must balance utility, fairness, and equity under uncertainty (14). Model outputs may therefore shape not only prognosis, but access to a life-sustaining organ. Recipient-centric models that omit prognostic scoring, procurement quality, ischemic exposure, or organ-specific risk may overestimate suitability and favour recipients with stronger chart profiles. Biological suitability and allocation are thus inseparable: models estimating benefit may also influence access to scarcity. Transplant FMs must therefore treat paired biology, donor organ quality, cross-system linkage, and allocation under scarcity as coupled objectives.

### 2.3 Multimodal nature of transplant data

Transplantation is by nature multidisciplinary and multimodal, involving the expertise of surgery, nephrology, hepatology, cardiology, infectious disease medicine, psychology, and many more fields. Transplant modalities include the entire spectrum from clinical observations to laboratory studies, imaging, pathology, immunology, molecular diagnostics, pharmacologic and physiological studies, among others. These modalities can be characterized into clinical, imaging, biological, and longitudinal (15), but are still scattered between providers and systems (13). The challenge here is not simply how to fuse heterogeneous sources of data, but rather to interpret each modality in relation to the others and to the clinical state in which it was acquired.

Existing multimodal medical FMs often assume that relevant modalities are co-present and similarly informative at a single timepoint. Transplantation violates this assumption. Crossmatch results and organ-quality imaging are most pertinent pre-transplant, whereas donor-derived cell-free DNA, laboratory trajectories, and biopsy histology become more important during post-transplant surveillance. Pertinent modalities are also organ-dependent, with Banff histology in kidney transplantation, cardiac allograft vasculopathy imaging in heart transplantation, and vascular or bile duct evaluation in liver transplantation. Because these data arrive asynchronously, in heterogeneous formats, and with variable completeness, transplant FMs require a changing hierarchy of evidence and cross-modal clinical reasoning rather than fixed fusion or independent prediction on each stream (Supplementary Figure S2).

## 3 Foundation models in transplantation: current progress and remaining gaps

### 3.1 Current foundation model applications in transplantation

To date, published work has largely adapted general FMs to isolated transplant tasks rather than pretraining models on donor–recipient–graft trajectories across the transplant continuum. What the field has begun to explore is application by repurposing existing FMs for chest CT, pathology, tabular prediction, clinical language, or operative settings for problems such as donor–recipient matching, eligibility assessment, operative planning, graft-loss prediction, and rejection subtyping (16–21). These studies demonstrate component feasibility, but they have not yet demonstrated the integrated, transferable capabilities proposed here for transplant-native foundation modeling. Many address a single care phase, usually a single modality, and adapt a general model without transplant-native pretraining. We are not aware of models that learn a joint donor–recipient representation, propagate information across phase transitions, or embed allocation constraints into model design. Early LLM and copilot applications in eligibility assessment or documentation without donor-side grounding illustrate the adaptation trap: strong language performance on recipient charts does not establish transplant readiness (17, 22, 23).

These applications nonetheless point to what future transplant FMs would need to do differently. Imaging FMs can support donor–recipient matching and operative planning (16, 18), but only when linked to graft outcomes and procurement context rather than used as standalone segmentation or sizing tools. Pathology models may aid rejection subtyping (21), yet graft surveillance requires biopsy findings to be integrated with longitudinal laboratory, immunological, and treatment data. Tabular and FM approaches can support outcome prediction (20), yet simpler models may match or exceed them on some metrics when interpretability and deployment matter, so adoption must be task-justified, not assumed by default. Collectively, these studies establish component feasibility, but not yet integrated, transplant-native foundation modeling across the care continuum.

### 3.2 Why generalist medical foundation models fall short

Most medical AI, including today’s FMs, inherits a generalist predictive worldview (Supplementary Figure S3). It assumes that one patient can be modeled alone, that risk changes gradually, that a model can optimize one outcome at one timepoint, that large homogeneous datasets will suffice, and that learning correlations from observational data is enough. Transplant care challenges these assumptions. An organ offer links two clinical histories; physiology resets at implantation; a decision today reshapes risk tomorrow; cohorts are small and spread across centers; and treatment itself (immunosuppression, rejection therapy, and infection management) changes the trajectory the model is trying to predict. Generalist AI may perform well on the benchmark tasks illustrated on the left side of Supplementary Figure S3, but such performance alone does not establish that a model captures the linked donor, recipient, and graft trajectories needed for transplant care (24–26).

Clinical fine-tuning can improve performance on individual transplant tasks, but the broader challenge is architectural: models must be designed to connect information across records, modalities, and phases of care (Table 1). A language model fine-tuned to summarize clinic notes may still lack the design needed for organ offers, committee deliberations, or donor histories. An EHR model adapted to extract facts from one record may still struggle to reconcile recipient charts with HLA laboratories and allocation systems. An imaging model tuned for segmentation or classification may still fail to distinguish whether a post-transplant abnormality reflects rejection, infection, ischemia, or chronic change without timing and surrounding evidence. A pathology model adapted from oncology may still fall short of allograft injury interpretation tied to Banff criteria, donor-specific antibodies, and serial biopsy change. Multimodal systems fine-tuned on co-present inputs at one visit may still leave open how asynchronous, incomplete evidence should be weighted across care stages. Task-level adaptation can therefore demonstrate feasibility without producing a stack built for allocation-adjacent decisions under fairness and safety constraints, or for governed simulation of evolving graft risk without overstepping biopsy-based judgment.

**Table 1:** Conceptual transition from general medical FMs to transplant-native FMs.

FM capability	Current models	Transplant gap	Transplant-native target
Clinical language	Summarize notes, answer questions, and support dialogue in controlled settings (8, 45, 46).	Limited transplant terminology, fragmented documentation, and multi-team workflow context (22, 47, 48).	Transplant-aware reasoning over organ offers, committee notes, donor histories, and follow-up trajectories (4).
EHR/biomedical text	Clinical NLP, information extraction, and medical question answering (49–51).	Data span donor records, recipient EHRs, HLA labs, allocation platforms, and longitudinal follow-up (3, 48).	Longitudinal donor–recipient language models across rejection, infection, immunosuppression, and graft function (4).
Medical imaging	Segmentation, classification, report generation, and visual question answering (52–54).	General-purpose imaging models may miss the transplant-native significance of imaging findings (55).	Context-aware transplant imaging integrating graft anatomy, donor quality, serial imaging, and pathology (56–58).
Pathology	Whole-slide cancer detection, subtyping, and image–text retrieval (59–61).	Many pathology FMs have been developed around oncology-based tasks and may not capture transplant-native pathological findings (60, 62)	Allograft pathology FMs combining Banff scoring, DSA, molecular signatures, and biopsy evolution (21, 63, 64).
Multimodal FMs	Combine imaging, text, and pathology for retrieval and clinical reasoning (65–67).	Rarely encode paired donor–recipient biology, allocation ethics, or long-term immunosuppression.	Multimodal donor–recipient FMs jointly representing organ quality, immune compatibility, and outcomes.
Decision support	Diagnostic support, triage, and clinician-facing interaction (45, 46).	Isolated-task performance does not establish safety or equity when errors affect organ allocation (14, 22, 68, 69).	Bounded decision-support systems with silent trials, calibration, subgroup fairness, and monitoring.
Trajectory modelling and simulation	Simulation aids and retrospective trajectory modelling at limited timepoints (7, 24).	Transplant risks evolve through immunosuppression, rejection, infection, and treatment response (9, 12).	Digital transplant twins as bounded simulation aids for graft trajectories and immunosuppression, not substitutes for biopsy-based rejection calls or allocator judgment

General medical FMs remain insufficient for transplantation’s donor–recipient, longitudinal, and allocation-aware requirements.

## 4 From data to deployment: what transplantation needs from foundation models

Transplantation does not need more proof that generalist AI can be pressed into service at isolated points along the pathway. It needs models whose training objective, data architecture, and deployment guardrails match the clinical problem from the outset. Without broader evaluation across the care continuum, progress may remain a collection of promising subtasks rather than a coherent foundation for transplant care. The roadmap below outlines what this shift would require (Supplementary Figure S4).

### 4.1 Data infrastructure

Data silos, fragmentation, and lack of standardization of data continue to form essential obstacles in the creation of transplant FMs. The field has a number of useful registries such as OPTN (27), SRTR (28), CORR (29), CTR (30), and CanODT (31), but these have been established mainly for allocation purposes, monitoring and outcome measurement and not for the fine details and multimodal data necessary for training transplant FMs (32–34). National registries and multi-centre consortia should therefore lead the development of minimum datasets, common data elements, and standardized outcomes. Standardization should preserve timing, provenance, completeness, collection methods, and informative missingness. Priorities should progress from harmonized donor–recipient–graft datasets and provenance metadata, to interoperable and temporally aligned data exchange using SNOMED CT, LOINC, and HL7 FHIR, and ultimately to privacy-preserving federated learning and multi-site evaluation without centralizing raw records (35–40).

### 4.2 Model development

The next generation of transplant FMs should be designed around transplant-native clinical structure rather than relying solely on adaptation of generalist medical systems. That will require domain-adaptive pretraining, instruction tuning, retrieval-augmented reasoning, and multimodal continual learning on transplant-native data. The next frontier of models will have to learn joint representations that link organ quality, immune compatibility, surgical events, immunosuppression and patient trajectories (41, 42). Retrieval-augmented approaches may be particularly important for grounding outputs in guidelines, protocols, up-to-date evidence, de-identified clinical notes, and data from individual patients, guided by knowledge of surgery, pathology, immunology, infectious disease, pharmacology and ethics. Dynamic transplant models built on counterfactual reasoning may allow clinicians to simulate alternative treatment, timing, or allocation decisions, asking what graft trajectory would follow a different immunosuppression regimen, organ offer, or intervention timing, rather than only forecasting a single default trajectory, supporting graft surveillance and immunosuppressive optimization (43), but must have an assistive and not substitutive role. Although observational transplant data pose challenges for causal inference, including confounding, incomplete measurement, and limited data on some alternative decisions, dynamic models may still provide a useful framework for exploring alternative trajectories. Such simulations require rigorous validation before they can be used to augment clinical care.

### 4.3 Clinical evaluation and validation

Transplant FMs must be safe, fair, and reliable across the full trajectory, not merely accurate on retrospective benchmarks. Evaluation should extend beyond AUROC to calibration, uncertainty quantification, subgroup fairness across race and ethnicity, geography, organ type, pediatric vs. adult populations, and insurance or access patterns, workflow integration, and comparison against clinical standards (44). In the short term, evaluation should emphasize multicentre retrospective benchmarking using temporally ordered cases that reflect incomplete and asynchronous clinical data at real decision points. Mid-term evaluation should extend to silent trials, clinician-in-the-loop assessment, pragmatic multicentre studies, and measurement of clinical impact, including whether models improve decision-making without increasing safety risks, disparities, or workflow burden. Long-term validation will require ongoing monitoring for model drift, hallucinations and unsafe recommendations, clinical outcomes, and cost-effectiveness.

### 4.4 Deployment and governance

Because transplant FM outputs can shape access to a scarce, life-sustaining organ, human-in-the-loop oversight is not an optional safeguard layered on top of deployment but a structural requirement of it: final adjudication over allocation, matching, and treatment decisions must remain with the clinical team, with the model’s role bounded to surfacing evidence, flagging uncertainty, and supporting rather than replacing that judgment. Given the potential impact of transplant FMs on organ allocation, organ matching, immunosuppressive therapy, and management of transplanted organs, near-term introduction should be supervised and used in a supporting role to prepare committees, summarize organ offers, and triage transplant surveillance with uncertainty visualization, clinician overrides, and audit trails (22). While clinical decision support systems have been traditionally static, FMs generate adaptive and context-specific recommendations and hence must be managed in a continuing manner by governance frameworks that include regulatory requirements, liability structures, post-marketing surveillance, stakeholder collaboration, and calibration and output monitoring. Over time, coordination efforts at both the national and international level should work towards developing standards concerning issues of international exchanges, privacy, updating models, global fairness and availability, and access in resource-constrained regions.

## 5 Discussion

This Perspective argues that transplantation should not be treated as simply another downstream application of general-purpose medical FMs. Its central contribution is to define the donor–recipient–graft ecosystem, rather than the individual patient or isolated task, as the organizing unit for transplant AI, and to identify the longitudinal, multimodal, decision-aware, and governance requirements that follow from this structure. Foundational components already exist at isolated points across the transplant continuum (16, 17, 20, 21), but transplant-native FMs remain a proposed direction rather than an established superior architecture. Whether such models ultimately outperform well-designed task-specific or adapted general medical models will require rigorous comparative evaluation.

The immediate priorities are therefore not model scale alone, but harmonized donor–recipient–graft data, clinically meaningful multimodal representations, multicentre validation, calibration and uncertainty assessment, and carefully bounded evaluation within real clinical workflows. The place of FMs in transplantation will ultimately be determined where care actually happens: in committee rooms, operating theatres, and outpatient clinics, rather than on benchmarks alone. In these early stages, their role is to augment rather than to replace clinical judgment. Their use should remain clinically governed wherever model outputs inform organ allocation, graft surveillance, or treatment decisions. The models worth building are those that respect transplant biology, fit into current practice, protect patients, and promote fair and equitable access to scarce organs.

## Data Availability

The original contributions presented in the study are included in the article/Supplementary Material, further inquiries can be directed to the corresponding authors.
