# Supplementary material for: Beyond generalist AI: purpose-built foundation models for transplantation
Source: Front Med (Lausanne). 2026 Oct 21;13:1988867. doi: 10.3389/fmed.2026.1988867 (PMC13730831; doi:10.3389/fmed.2026.1988867)
Supplement: Supplementary file 1 [file Datasheet1.pdf]

Supplementary Material: Beyond Generalist AI: Toward Purpose-Built Foundation Models for Transplantation

1 SUPPLEMENTARY FIGURES

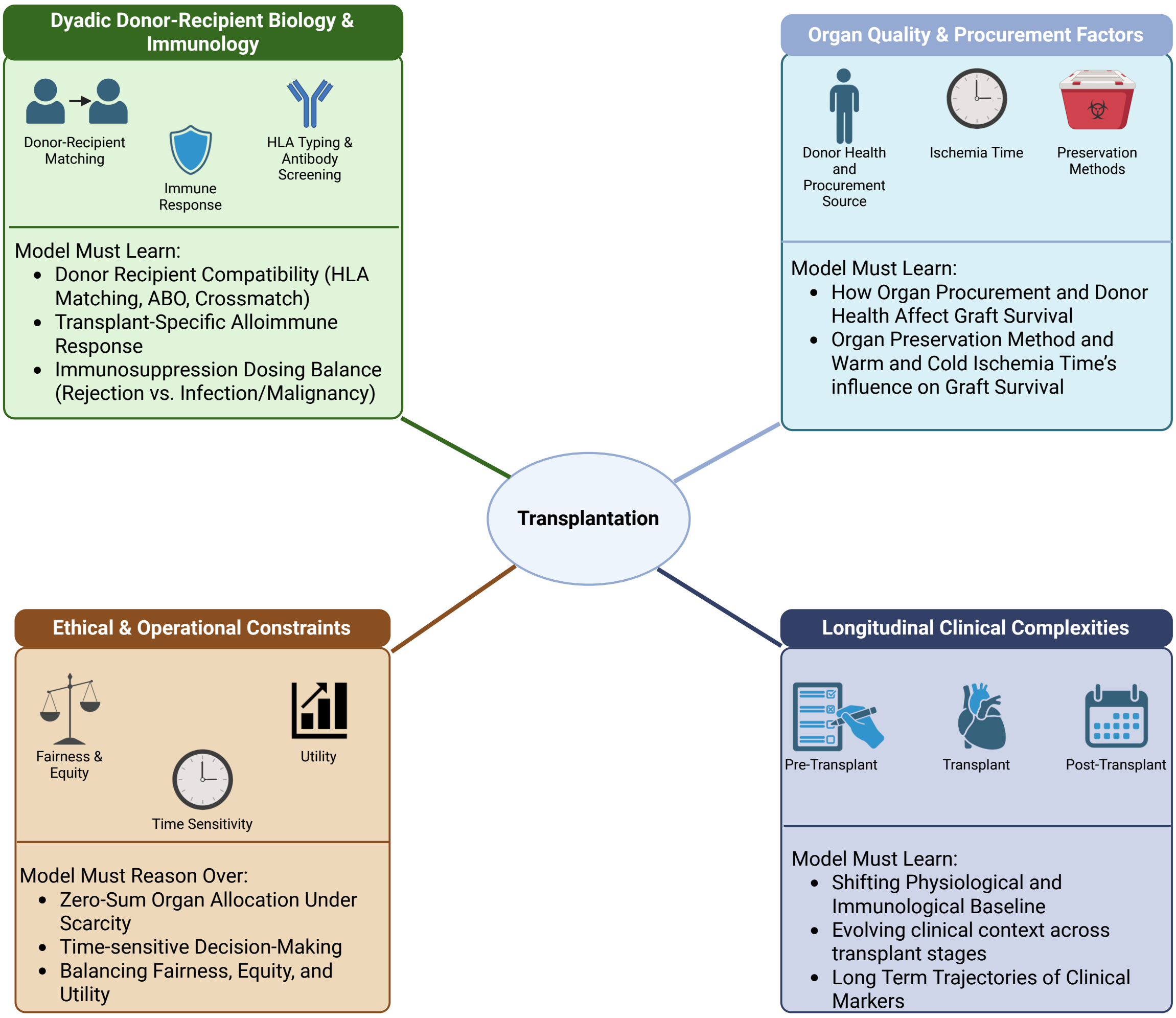

Figure S1. Supplementary Figure S1. What makes transplant unique. Key characteristics of transplantation that present unique challenges and opportunities for designing transplant-specific FMs. For each category, selected requirements for what an FM must learn or reason over are highlighted. Created in BioRender. K, V. (2026) https://BioRender.com/n8a4jtn

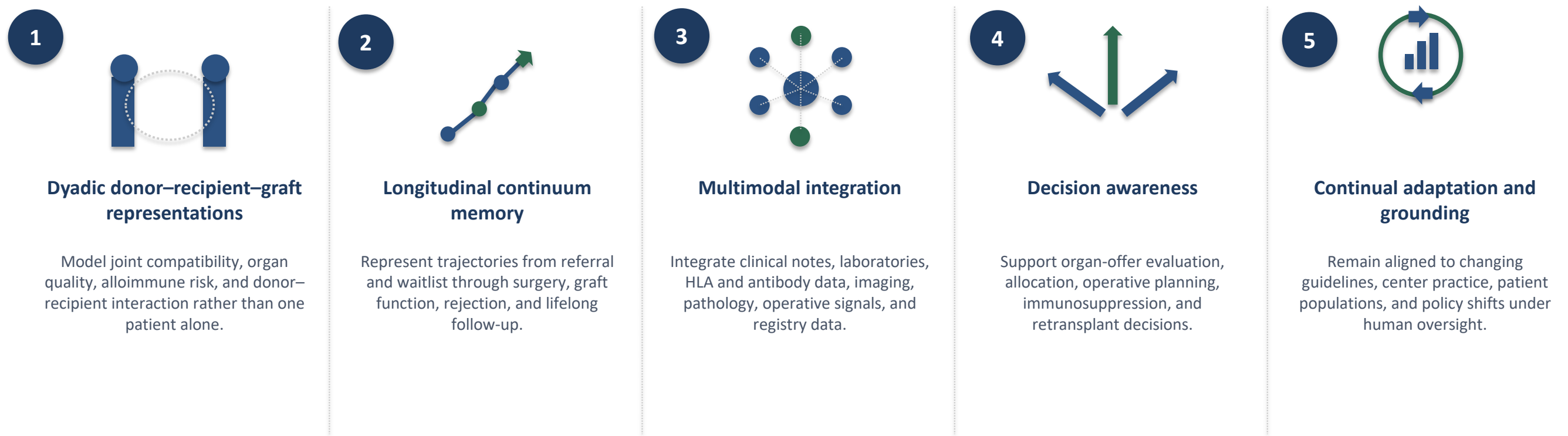

Figure S2. Supplementary Figure S2. What a transplant FM must learn. Five core requirements for transplant-specific FMs: dyadic donor–recipient–graft representations, longitudinal continuum memory, multimodal integration, decision awareness, and continual adaptation and grounding under human oversight.

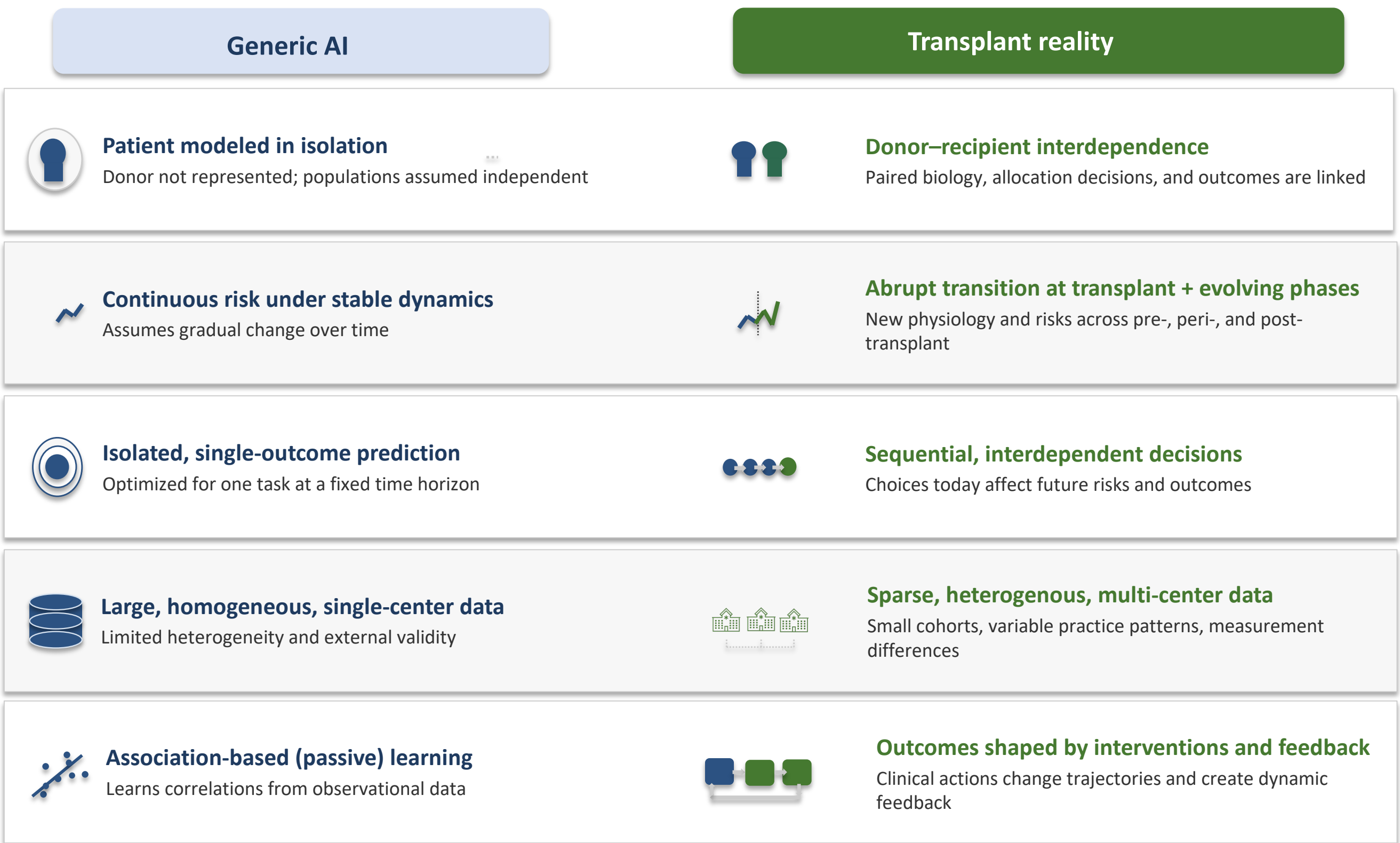

Figure S3. Supplementary Figure S3. Why generalist predictive AI is insufficient for transplantation. Contrasts between assumptions of generalist predictive AI and the clinical realities of transplant care, including donor–recipient interdependence, phase transitions, sequential decisions, sparse multi-center data, and intervention-driven feedback.

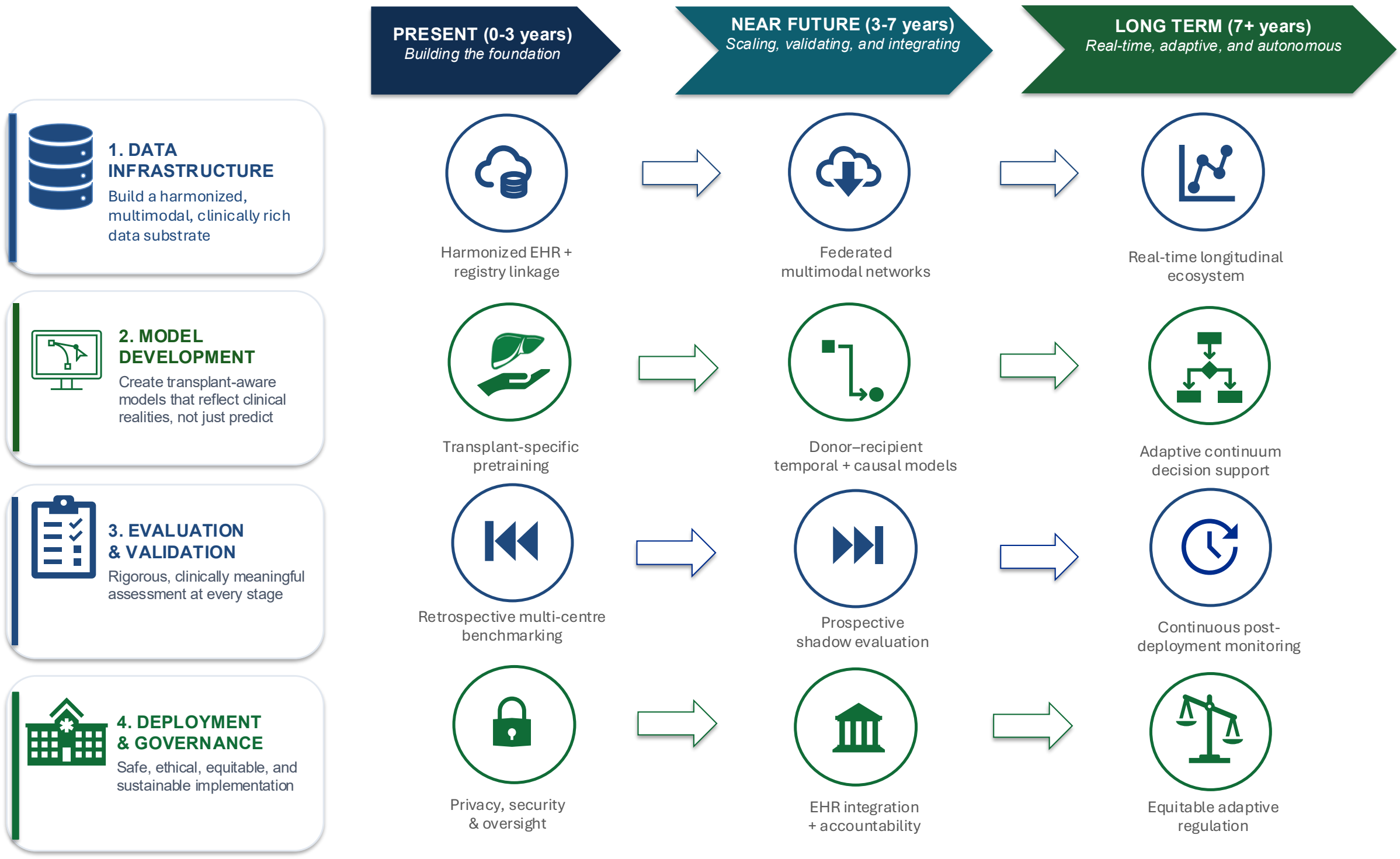

Figure S4. Supplementary Figure S4. FMs in transplantation: a staged roadmap. Priorities across data infrastructure, model development, evaluation and validation, and deployment and governance from present (0–3 years) through near future (3–7 years) to long term (7+ years), with governed, assistive deployment in the long term.

2 SUPPLEMENTARY TABLE

Table S1. Supplementary Table S1. Selected clinical objectives across the transplant continuum: technical, risk, and safety considerations. Clinical objectives across pre-transplant, perioperative, and post-transplant stages where FMs may be applied, with key data modalities, technical considerations, and risk and safety considerations. **Bold objectives** denote highest-value, highest-risk FM targets emphasized in the text.

Transplant Stage	Clinical Objectives	Key Modalities	Technical Considerations	Risk and Safety Considerations
Pre-Transplant	**Donor-Recipient Matching and Organ Allocation** [17]	Immune Profiling (HLA typing, crossmatching, ABO typing), Laboratory Data, Clinical Data and Notes, Imaging, Prognostic Scoring (MELD, KDPI), Donor Procurement and Quality Data, Waitlist Data, Psychosocial Assessment	Integration of multimodal, fragmented, cross-institutional donor-recipient data. Reasoning under incomplete and time-sensitive information.	Accurate compatibility matching is needed to ensure graft survival and minimize risk of rejection. Organ allocation must balance fairness, equity, and utility.
	Waitlist Prioritization [18]	Clinical Notes and Data, Prognostic Scoring, Laboratory Data, Imaging, Waiting Time	Longitudinal modelling of evolving patient state on the transplant waitlist.	Fair prioritization and equitable access.
	Risk Stratification	Clinical Data, Immune Profiling, Pathology, Imaging, Psychosocial Assessment	Integration of multimodal, multi-specialty, and fragmented data.	Inaccurate risk estimates may lead to inappropriate candidate selection or missed contraindications.
Perioperative	Operative Planning [19]	Imaging, Clinical Data, Laboratory Data	Integration of multimodal data for patient-specific anatomical and surgical planning.	Inaccurate donor organ or patient assessment may lead to intraoperative complications or organ sizing mismatch.
	Intraoperative Decision Making [20]	Physiological Monitoring, Real-Time Imaging, and Intraoperative Sensing and Tracking (surgical video, instrument tracking, environmental sensors)	Real-time integration of multimodal data across rapidly changing physiological conditions.	Timely detection of critical events such as hemodynamic instability is essential to prevent adverse intraoperative outcomes.
	Immediate Post-Operative Management	Physiological Monitoring, Laboratory Data, Imaging, Graft Biopsy	Rapid integration of multimodal post-operative data to detect early graft dysfunction and complications in a time-critical window.	Timely detection of post-operative complications such as primary graft dysfunction, delayed graft function, and early allograft dysfunction is essential to prevent graft loss.
Post-Transplant	**Graft Function and Rejection Surveillance** [21, 22]	Graft Biopsy, Histopathology, Imaging, Molecular Biomarkers (dd-cfDNA), Immunological Data	Longitudinal monitoring across heterogeneous and multidisciplinary data sources over the patient's lifetime.	Timely detection of graft rejection or dysfunction is critical for long-term graft and patient survival.
	Immunosuppression Optimization	Drug levels (e.g., Trough Concentrations), Longitudinal Clinical Data, Laboratory Values	Temporal modelling of treatment response and biomarker trajectories.	Improper immunosuppression management can lead to infections and malignancies (oversuppression) or rejection (undersuppression).
